# Supplementary material for: Investigation of High Frequency Irreversible Electroporation for Canine Spontaneous Primary Lung Tumor Ablation
Source: Biomedicines. 2024 Sep 7;12(9):2038. doi: 10.3390/biomedicines12092038 (PMC11428908; doi:10.3390/biomedicines12092038)
Supplement: Supplementary file 1 [file biomedicines-12-02038-s001.zip › supplemental table S1 gene table.pdf]

Gene Symbol	Gene Name	Refseq
ACKR3	Chemokine (C-X-C motif) receptor 7	NM_001003281
AICDA	Activation-induced cytidine deaminase	NM_001003380
BCL2	B-cell CLL/lymphoma 2	NM_001002949
BCL2L1	BCL2-like 1	NM_001003072
CCL2	Chemokine (C-C motif) ligand 2	NM_001003297
CCL20	Chemokine (C-C motif) ligand 20	NM_001005254
CCL28	Chemokine (C-C motif) ligand 28	NM_001005257
CCL4	Chemokine (C-C motif) ligand 4	NM_001005250
CCL5	Chemokine (C-C motif) ligand 5	NM_001003010
CCR1	Chemokine (C-C motif) receptor 1	NM_001038606
CCR10	Chemokine (C-C motif) receptor 10	XM_844228
CCR2	C-C motif chemokine receptor 2	XM_005632630
CCR4	Chemokine (C-C motif) receptor 4	NM_001003020
CCR5	Chemokine (C-C motif) receptor 5	NM_001012342
CCR7	Chemokine (C-C motif) receptor 7	XM_548131
CCR9	Chemokine (C-C motif) receptor 9	NM_001284476
CD274	CD274 molecule	XM_005615931
CSF1	Colony stimulating factor 1 (macrophage)	XM_005621807
CSF2	Colony stimulating factor 2 (granulocyte-macrophage)	NM_001003245
CSF3	Colony stimulating factor 3 (granulocyte)	XM_005624543
CTLA4	Cytotoxic T-lymphocyte-associated protein 4	NM_001003106
CXCL10	Chemokine (C-X-C motif) ligand 10	NM_001010949
CXCL11	Chemokine (C-X-C motif) ligand 11	XM_003640114
CXCL12	Chemokine (C-X-C motif) ligand 12	NM_001128097
CXCL5	Chemokine (C-X-C motif) ligand 5	XM_849650
CXCR1	Chemokine (C-X-C motif) receptor 1	XM_005640640
CXCR2	Interleukin 8 receptor, beta	NM_001003151
CXCR3	Chemokine (C-X-C motif) receptor 3	NM_001011887
CXCR4	Chemokine (C-X-C motif) receptor 4	NM_001048026
CXCR5	Chemokine (C-X-C motif) receptor 5	XM_546496
EGF	Epidermal growth factor	NM_001003094
EGFR	Epidermal growth factor receptor	XM_014120756
FOXP3	Forkhead box P3	NM_001168461
GZMA	Granzyme A (granzyme 1, cytotoxic T-lymphocyte-associated serine esterase 3)	XM_544335
GZMB	Granzyme B	XM_547752
HIF1A	Hypoxia inducible factor 1, alpha subunit (basic helix-loop-helix transcription factor)	NM_001287163
IDO1	Indoleamine 2,3-dioxygenase 1	XM_532793
IFNG	Interferon gamma	NM_001003174

IGF1	Insulin-like growth factor 1 (somatomedin C)	NM_001313855
IL10	Interleukin 10	NM_001003077
IL12A	Interleukin 12A (natural killer cell stimulatory factor 1, cytotoxic lymphocyte maturation factor 1, p35)	NM_001003293
IL12B	Interleukin 12B (natural killer cell stimulatory factor 2, cytotoxic lymphocyte maturation factor 2, p40)	NM_001003292
IL13	Interleukin 13	NM_001003384
IL15	Interleukin 15	NM_001197188
IL17A	Interleukin 17A	NM_001165878
IL1A	Interleukin 1, alpha	NM_001003157
IL1B	Interleukin 1, beta	NM_001037971
IL1R1	Interleukin 1 receptor, type I	XM_538449
IL2	Interleukin 2	NM_001003305
IL22	Interleukin 22	XM_538274
IL23A	Interleukin 23, alpha subunit p19	XM_538231
IL4	Interleukin 4	NM_001003159
IL5	Interleukin 5 (colony-stimulating factor, eosinophil)	NM_001006950
IL6	Interleukin 6 (interferon, beta 2)	NM_001003301
IRF1	Interferon regulatory factor 1	XM_538621
MYC	V-myc myelocytomatosis viral oncogene homolog (avian)	NM_001003246
MYD88	Myeloid differentiation primary response gene (88)	XM_534223
NFKB1	Nuclear factor of kappa light polypeptide gene enhancer in B-cells 1	NM_001003344
NOS2	Nitric oxide synthase 2, inducible	NM_001003186
PDCD1	Programmed cell death 1	XM_543338
PTGS2	Prostaglandin-endoperoxide synthase 2 (prostaglandin G/H synthase and cyclooxygenase)	NM_001003354
SPP1	Secreted phosphoprotein 1	XM_003434023
STAT1	Signal transducer and activator of transcription 1, 91kDa	XM_843260
STAT3	Signal transducer and activator of transcription 3 (acute-phase response factor)	XM_548090
TGFB1	Transforming growth factor, beta 1	NM_001003309
LAMP1	Toll-like receptor 2	NM_001005264
TLR2		
TLR3	Toll-like receptor 3	XM_540020
TLR4	Toll-like receptor 4	NM_001002950
TLR7	Toll-like receptor 7	NM_001048124
TLR9	Toll-like receptor 9	NM_001002998
TNF	Tumor necrosis factor	NM_001003244

IL18	Interleukin 18 (interferon-gamma-inducing factor)	NM_001003169
FASLG	Fas ligand (TNF superfamily, member 6)	NM_001287153
CXCL8	Interleukin 8	NM_001003200
APLNR	Apelin receptor	XM_005631209
JAK1	Janus kinase 1	NM_001287126
JAK2	Janus kinase 2	XM_541301
ICAM1	Intercellular adhesion molecule 1	NM_001003291
IL2RA	Interleukin 2 receptor, alpha	NM_001003211
CD244	CD244 molecule, natural killer cell receptor 2B4	XM_014111156
CCL13	Chemokine (C-C motif) ligand 13	NM_001003966
CD209	CD209 molecule	NM_001130832
TBX21	T-box 21	XM_548164
NCR1	Natural cytotoxicity triggering receptor 1	NM_001284448
IL21R	Interleukin 21 receptor	XM_005621391
IL1R2	Interleukin 1 receptor, type II	XM_005626003
PDCD1LG2	Programmed cell death 1 ligand 2	XM_005615944
TNFRSF4	Tumor necrosis factor receptor superfamily, member 4	XM_546720
HPRT1	Hypoxanthine phosphoribosyltransferase 1	NM_001003357
GAPDH	Glyceraldehyde-3-phosphate dehydrogenase	NM_001003142
ACTB	Actin, beta	NM_001195845
