# Supplementary figures and images for: Investigation of High Frequency Irreversible Electroporation for Canine Spontaneous Primary Lung Tumor Ablation
Source: Biomedicines. 2024 Sep 7;12(9):2038. doi: 10.3390/biomedicines12092038 (PMC11428908; doi:10.3390/biomedicines12092038)

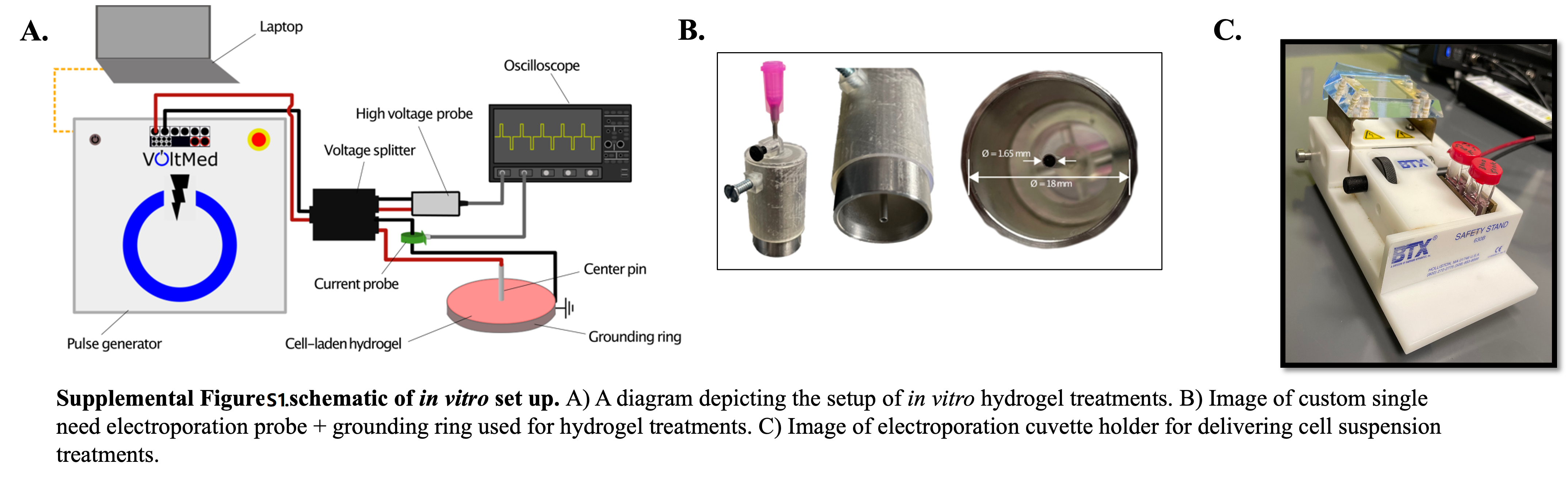

Supplement: Supplementary file 1 [file biomedicines-12-02038-s001.zip › Supplemental figure S1.png]

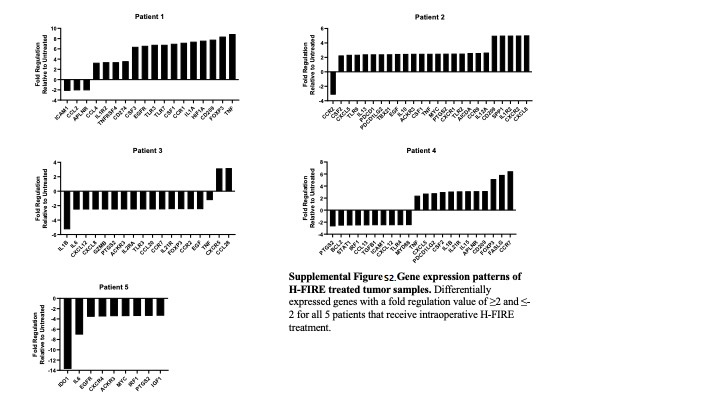

Supplement: Supplementary file 1 [file biomedicines-12-02038-s001.zip › Supplemental figure S2.jpg]

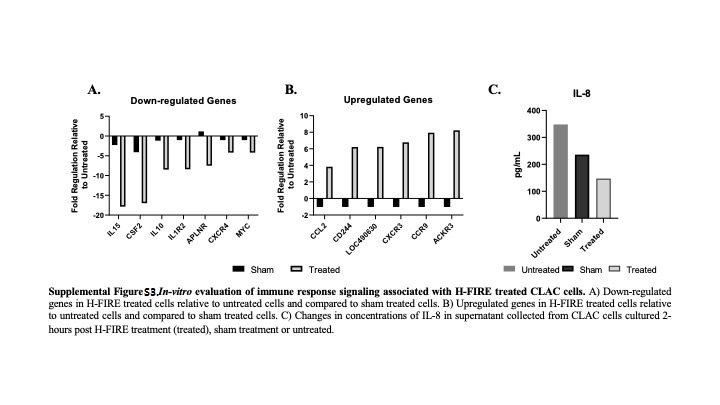

Supplement: Supplementary file 1 [file biomedicines-12-02038-s001.zip › supplemental figure S3.jpg]
